# Utilization of PEGylated cerosomes for effective topical delivery of fenticonazole nitrate: *in-vitro* characterization, statistical optimization, and *in-vivo* assessment

**DOI:** 10.1080/10717544.2020.1859000

**Published:** 2020-12-16

**Authors:** Rofida Albash, Carol Yousry, Abdulaziz Mohsen Al-Mahallawi, Ahmed Adel Alaa-Eldin

**Affiliations:** a Department of Pharmaceutics, College of Pharmaceutical Sciences and Drug Manufacturing, Misr University for Science and Technology, Giza, Egypt; b Department of Pharmaceutics and Industrial Pharmacy, Faculty of Pharmacy, Cairo University, Cairo, Egypt; c Department of Pharmaceutics, Faculty of Pharmacy, October University for Modern Sciences and Arts (MSA), Giza, Egypt; d Department of Pharmaceutics, Faculty of Pharmacy, Fayoum University, Elfayoum, Egypt

**Keywords:** Fenticonazole nitrate, Brij^®^, PEGylated cerosomes, Ceramide, dermatokinetic study, histopathological study

## Abstract

In this investigation, we focused on ceramide IIIB, a skin component whose depletion tends to augment multiple skin disorders and fungal infections. Ceramide IIIB was included into PEGylated surfactant-based vesicular phospholipid system to formulate ‘PEGylated cerosomes’ (PCs) loaded with fenticonazole nitrate (FTN). FTN is a potent antifungal agent adopted in the treatment of mixed mycotic and bacterial infections. The ceramide content of the vesicles may provide protective and regenerative skin activity whereas Brij^®^; the PEGylated surfactant, can enhance drug deposition and skin hydration. Both components are expected to augment the topical effect of FTN. PCs were prepared by thin-film hydration technique. A 2^3^ full-factorial design was applied to study the effect of ceramide amount (X_1_), Brij type (X_2_) and Brij amount (X_3_) on the physicochemical properties of the formulated PCs namely; entrapment efficiency (EE%;Y_1_), particle size (PS;Y_2_), polydispersity index (PDI;Y_3_) and zeta potential (ZP;Y_4_). The optimal formula was selected for further *in-vivo* dermatokinetic and histopathological study. The optimal FTN-loaded PC (PC6) showed nanosized cerosomes (551.60 nm) with high EE% (83.00%w/w), and an acceptable ZP value of 20.90 mV. Transmission electron micrographs of the optimal formula illustrated intertwined tubulation form deviated from the conventional spherical vesicles. Finally, the dermatokinetic study of PC6 showed higher drug concentration and localization of FTN in skin layers when compared with FTN suspension and the histopathological study confirmed its safety for topical application. The overall findings of our study verified the effectiveness of utilizing PEGylated cerosomes to augment the activity of FTN as a topical antifungal agent.

## Introduction

Skin infections, triggered by different fungal species such as *Candida albicans* and *Trichophyton* species, have been spread worldwide recently. Fungal infections are more frequently occurring due to the increase in the number of immunocompromised patients due to cancer chemotherapy, organ transplantation and human immunodeficiency virus infections (Veraldi & Milani, 2008). They are mainly treated by systemic oral administration and/or topical application of antifungal agents. Although oral administration of systemic antifungal agents is known to be more effective, it usually results in toxic side effects and increased risk of drug-drug interaction (Abd-Elsalam et al. 2018). Fenticonazole nitrate (FTN) is an antifungal imidazole derivative that acts by inhibiting ergosterol synthesis and consecutively damaging the cytoplasmatic membrane (Campos et al., 2018). It also blocks cytochrome oxidases and peroxidases and specifically inhibits the secretion of protease acid by *Candida albicans* which promotes the yeast adherence to the epithelial cells (Veraldi & Milani, 2008). Thus, FTN has fungistatic and fungicidal activities on dermatophytes, yeasts and fungi. In addition, FTN exhibits a broad-spectrum antibacterial activity that includes Gram positive bacteria and bacteria commonly associated with fungal skin and vaginal infections (Jung et al., 1988). Therefore, FTN is considered an ideal topical agent for mixed mycotic and bacterial infections, alternative to other multiagent treatments (Veraldi & Milani, 2008).

Ceramides are the simplest and the most hydrophobic type of sphingolipids, which are responsible for the barrier function of the stratum corneum (SC). Ceramides represent 50% of the lipid weight of the SC, however, they are present in a much lower proportion in cell membranes (Khazanov et al., 2008). Some reports support that ceramide-containing formulations, at an optimum concentration of 0.05% w/w; may aid in the renewal of the skin’s natural protective layer and formation of an effective barrier against moisture loss. This is particularly suitable for long-term protection and repair of sensitive and dry skin (Su et al., 2017). Several studies have been devoted to identify the different types of ceramides present in the human skin and their functionwhere they reported that alterations in ceramide content are associated with many skin diseases such as atopic dermatitis, psoriasis and topical fungal infections (Meckfessel & Brandt, 2014). A study conducted on mice showed that ceramide was essential for their survival. Mice deficient in ceramide died shortly after birth as a result of trans-epidermal water loss. Cultured skin from mice was also more susceptible to colonization by *Candida albicans*, emphasizing the importance of skin barrier function to protect against foreign insults (Jennemann et al., 2012).

Cerosomes are ceramide-enclosed tubulated vesicles formulated using different surfactants and phospholipids for pharmaceutical application. Such carriers allow good incorporation and dissolution of ceramide. They also offer good skin tolerability, permeability and high drug bioavailability when applied topically. The surfactant addition in the formulation aids in the production of highly stable unaggregated vesicles of the double lipidic, phosphatidylcholine-ceramide mixture. Abdelgawad et al. (2017) previously reported the successful application of cerosomes for the topical delivery of tazarotene for the treatment of psoriasis.

Here in the present work, we investigated the applicability, efficacy, and safety of PEGylated Cerosomes (PCs) as a novel FTN-carrier system for the effective topical treatment of fungal infections. The incorporation of ceramide within the system may augment the effect of FTN through the regeneration of the skin’s natural protective layer and the restoration of its normal ceramide content. Brij^®^ was utilized as PEGylated surfactant to prepare PEGylated cerosomes (PCs). Brij^®^ consists of PEGylated single-chain surfactant with various PEG chain lengths and acyl chain entities (Tagami et al., 2011). The presence of the hydrophilic PEG moieties could enhance the water uptake in SC which leads to SC swelling and probable structural alterations in its lipids content and/or corneocytes which may increase the skin hydration subsequently (Rangsimawong et al., 2014). Besides, PEGylation of the formulated vesicles may modulate the interfacial properties of the vesicles which might influence their deposition and prolong their residence time at the site of action. Prolonged residence of the vesicles on the skin will improve the efficacy of topical drug administration as it will maintain the desired drug concentration over a prolonged period in the skin (Vega et al., 2013). To the best of our knowledge, this study is the first to investigate the applicability of Cerosomes as a drug carrier for enhanced topical treatment of fungal infections and the first to study the effect of PEGylated surfactant incorporation in cerosome formulation.

For the optimization of PC formulation, a 2^3^ full factorial design was adopted to study the effect of different variables influencing vesicles’ physicochemical parameters and select the most desirable formula. Ceramide amount (X_1_), Brij type (X_2_) and Brij amount (X_3_) were studied as independent variables, while entrapment efficiency (EE%; Y_1_), particle size (PS; Y_2_), polydispersity index (PDI; Y_3_) and zeta potential (ZP; Y_4_) were selected as the dependent variables. The optimal PC was evaluated for its morphology. Finally, a dermatokinetic and histopathological study was conducted in male Wistar rats to evaluate the efficacy and safety of the optimal FTN-loaded PC system.

## Materials

Fenticonazole nitrate (FTN) was supplied as a gift from Andalous Pharmaceutical Co. (Cairo, Egypt). L-α-phosphotidylcholine from egg yolk was purchased from Sigma Aldrich Chemical Co. (St. Louis, MO, USA). Stearylamine (SA) was obtained from Fluka Chemical Co. (Germany). Ceramide IIIB was kindly provided by Evonic Co. (Germany). Brij52 (polyoxyethylene (2) cetyl ether) and Brij97 (polyoxyethylene (10) oleyl ether) were provided by BASF Co. (New Jersy, NY, USA). Methanol and chloroform were obtained from El-Nasr Pharmaceutical Chemicals Co. (Cairo, Egypt).

## Methods

### Preparation of FTN-loaded PCs

PCs were prepared using thin-film hydration method (Albash et al., 2019). Briefly, phospholipid (100 mg), FTN (10 mg), SA as positive charge inducer (5 mg), in addition to variable amounts of ceramide IIIB and Brij^®^ (Brij52 or Brij97) were weighed in a long-necked round-bottom flask and dissolved in 10 mL chloroform: methanol (2:1;V/V) (Table 1). By maintaining pressure under vacuum for 30 min, the organic phase was slowly evaporated at 60 °C using a rotatory evaporator (Rotavapor, Heidolph VV 2000, Burladingen, Germany) at 90 rpm so that a thin clear film of PCs was formed. The film was thoroughly hydrated using 10 mL distilled water at 60 °C which is above the lipid phase transition temperature (Tc) for 45 min (Varona et al., 2011). The vesicles’ dispersion was left overnight at 4 °C to obtain mature vesicles.

**Table 1.** The levels of the independent variables in a 2^3^ full-factorial design for the evaluation of FTN-loaded PCs and the optimization criterion for the dependent variables.

Factors (independent variables)	Levels	
	Low	High
X_1_: Ceramide amount (mg)	15	30
X_2_: Brij type	Brij52	Brij97
X_3_: Brij amount (mg)	5	15
Responses (dependent variables)	Constraints	
Y_1_: EE (%)	Maximize	
Y_2_: PS (nm)	Minimize	
Y_3_: PDI	–	
Y_4_: ZP	Maximize	

EE%: entrapment efficiency percent; FTN: fenticonazole nitrate; PS: particle size; PDI: polydispersity index; ZP: zeta potential; PCs: PEGylated cerosomes.

### Characterization and optimization of FTN-loaded PCs

#### Determination of entrapment efficiency percentage (EE%)

The vesicular dispersion for the prepared formulae was centrifuged at 20,000 rpm for 1 h at 4 °C using a cooling centrifuge (Sigma 3 K 30, Germany). The sedimented vesicles were then lysed using methanol and analyzed spectrophotometrically at *λ*_max_ 252 nm using UV-Vis spectrophotometer (Shimadzu UV1650 Spectrophotometer, Koyoto, Japan). EE% was determined by using the following equation (Abdellatif et al., 2017):

(1) $$\text{EE}\%=(\frac{\text{Entrapped FTN amount}}{\text{Total FTN amount}})\times 100$$

All measurements were performed in triplicate.

#### Determination of particle size (PS), polydispersity index (PDI) and zeta potential (ZP)

The mean PS and PDI of the formulated vesicles’ dispersions were determined using a Malvern Zetasizer 2000 (Malvern Instruments Ltd., UK). The ZP evaluation was carried out by monitoring the electrophoretic movement of the particles in the electrical field. The measurements were performed after dilution (1:100) (Abd-Elsalam et al. 2018; Yousry et al. 2020). All measurements were performed in triplicate.

#### Experimental design construction and selection of the optimal FTN-loaded PCs

A 2^3^ full factorial design was constructed to evaluate the effect of 3 independent variables, each at 2 different levels, on PC formulation. The independent variables under investigation were ceramide amount (X_1_), Brij type (X_2_) and Brij amount (X_3_), while EE%, PS, PDI and ZP were selected as the dependent variables reflecting the vesicles’ characteristics as shown in Table 1. Eight different systems representing all possible combinations between factors were performed in duplicate.

Analysis of variance (ANOVA) test was performed for each parameter using Design expert^®^ software version 11 (Stat Ease, Inc., Minneapolis, MN, USA). *p*-values <0.05 were considered statistically significant.

System optimization was done to suggest the formula with the highest EE% and ZP values as well as the least PS and PDI. The suggestion was based on the desirability function which allows the investigation of all the constraints and responses at the same time and consequently, the formula with the highest desirability factor was selected. Finally, the suggested optimal formula was prepared, characterized, and compared with the predicted responses to confirm the accuracy of the model performance.

#### Transmission electron microscopy (TEM)

The morphology of the optimal PC was examined using a transmission electron microscope (Joel JEM 1230, Tokyo, Japan). A drop of the vesicular dispersion was placed as a thin film on a carbon-coated copper grid and stained with phosphotungstic acid solution (2% w/v). The sample was left to dry and examined using TEM at 80 kV (Yousry et al. 2020).

#### Short term physical stability study

The physical stability of the optimal PC was investigated to monitor the extent of vesicles’ growth, drug leakage or any other physical change. The optimal formula was stored at room temperature for 3 months and its stability was then evaluated by measuring and comparing the PS, PDI and EE% of the stored formula with the freshly prepared one (Farrah et al., 2020). In addition, the system was visually inspected for any particle aggregation or sedimentation.

#### *In-vivo* studies

Male Wistar rats, weighing 150–200 gm, with an average age of 7 weeks were used in the in vivo experimental studies. The use and handling of animals in all studies were in compliance with the EU directive 2010/63/EU for animal experiment and the study protocol was approved by Research Ethics Committee (REC) for experimental and clinical studies at Faculty of Pharmacy, Cairo University. The animals were kept in cages at a temperature of 22 °C and relative humidity of 55%. Water and rodent chow were provided. The animals were kept in a dark: light cycle of 12 hours each. Rats were left for 7 days for adaptation before experiments. A total of 36 animals were used in the dermatokinetic study while six animals were used in the histopathological study. For the topical administration of FTN suspension and optimal FTN-loaded PCs in the in vivo studies, bottle caps were utilized which acted as drug pools with an area of 4.91 cm2. The bottle caps were fixed to dorsal rat skin which was shaved with an electric clipper 24 hours before application of the samples. Moreover, the samples were added non-occlusive into the drug pools (Abdelbary & AbouGhaly, 2015).

#### Dermatokinetic study

Animals were divided into two groups of 18 animals each. One group received FTN suspension and the other received the optimal PC6 topically. Half milliliter of each sample (FTN suspension and optimal PCs) – equivalent to 0.5 mg – was applied to dorsal rat skin. After treatment, three animals from each group were sacrificed at different time intervals (1, 2, 4, 6, 8, and 10 hours). The animal carcasses were incinerated after excision of their skin. The excised skin was cut into pieces and sonicated in 5 mL methanol for 30 minutes. The extract was then filtered through a 0.45 μm filter and the concentration of FTN was determined by HPLC as previously reported by Quaglia et al. (2001) Dermatokinetic data was analyzed and the different dermatokinetic parameters such as *T*_max_, *C*_max_ and AUC_0–10_ were calculated using Kinetica^®^ 5 software (Thermo Fisher Scientific Inc., Waltham, MA, USA). *C*_max_ and AUC_0–10_ for both treatments were compared using one-way ANOVA statistical test. In addition, nonparametric signed-rank test (Mann–Whitney’s test) was employed to compare the medians of *T*_max_ for the two treatment groups using SPSS^®^ software 22.0. The difference at *p* < 0.05 was considered significant (Albash et al., 2019).

#### Histopathologic evaluation

*In-vivo* histopathological study was conducted to assess the irritation potential and observe the ultrastructural changes in the skin upon exposure to FTN-loaded PCs. The rats were randomly divided into 2 groups with 3 rats each. Group I acted as control while rats in group II were subjected to topical application of the optimal FTN-loaded PCs, three times daily for 1 week. The rats were then sacrificed, and the skin was excised for histopathological investigation. Autopsy samples were taken from the skin of rats in different groups, fixed in 10% formol saline for 24 hours, washed with tap water and then dehydrated using serial dilutions of alcohol (methyl, ethyl, and absolute ethyl). Specimens were cleared in xylene and embedded in paraffin at 56 °C in a hot air oven for 24 hours. Sections from the paraffin blocks of 4 mm thickness were cut using a microtome (Leica Microsystems SM2400, Cambridge, England). The obtained tissue sections were collected on glass slides, deparaffinized, stained with hematoxylin and eosin, and then examined under an electric light microscope (Abdelbary & AbouGhaly, 2015).

## Results and discussion

### Statistical evaluation of the factorial design

Design-expert^®^ software was used to study the different formulation variables in a 2^3^ full factorial design for the preparation of optimal FTN-loaded PCs. The developed design suggested 8 experimental runs representing all possible combinations of the different levels of the studied factors (Table 2). The dependent variables were analyzed according to two-factor interaction (2FI) model as it showed the highest prediction *R*^2^ value. The adequate precision value of the model is used to confirm its adequacy to navigate the design space (Albash et al. 2019). A ratio higher than four is preferred which was noticed for all the dependent variables as illustrated in Table 3. The adjusted and predicted *R*^2^ should be within approximately 0.20 of each other to represent a reasonable agreement. As shown in Table 3, the predicted *R*^2^ values were in good harmony with the adjusted *R*^2^ in all the dependent variables.

**Table 2.** Experimental runs composition corresponding to the 2^3^ full factorial design for FTN-loaded PC formulation and their resultant dependent variables.

	Ceramide amount (mg)	Brij type	Brij amount (mg)	EE%	PS (nm)	PDI	ZP (mV)
PC1	15	Brij 52	5	85.33 ± 1.24	458.00 ± 18.00	0.51 ± 0.03	18.50 ± 0.28
PC2	30			99.33 ± 0.48	630.00 ± 9.20	0.53 ± 0.05	11.00 ± 0.38
PC3	15	Brij 97		82.66 ± 1.25	336.6 ± 12.35	0.50 ± 0.06	16.70 ± 0.35
PC4	30			92.33 ± 1.25	422.00 ± 21.96	0.84 ± 0.03	2.74 ± 0.13
PC5	15	Brij 52	15	77.00 ± 0.81	309.20 ± 9.06	0.56 ± 0.03	14.30 ± 0.14
PC6	30			83.00 ± 1.63	551.60 ± 23.84	0.44 ± 0.05	20.90 ± 0.55
PC7	15	Brij 97		69.66 ± 0.47	361.50 ± 20.34	0.71 ± 0.07	2.80 ± 0.09
PC8	30			78.33 ± 2.86	392.50 ± 8.65	0.57 ± 0.02	7.92 ± 0.17

Data represented as mean ± SD (*n* = 3). EE%: entrapment efficiency percentage; FTN: fenticonazole nitrate; PS: particle size; PDI: polydispersity index; PC: PEGylated cerosomes.

**Table 3.** Output data of the 2^3^ full factorial design implemented for optimizing PCs formulations with the predicted and observed values for the optimal FTN-loaded PC (PC6).

Responses	EE%	PS (nm)	ZP (mV)
Adjusted *R*^2^	0.94	0.94	0.98
Predicted *R^2^*	0.89	0.90	0.978
Adequate precision	20.58	20.11	38.09
Significant factors	X_1_, X_2_, X_3_	X_1_, X_2_, X_3,_	X_1_, X_2_
Observed value of optimal formula (PC6)	83	551.6	20.9
Predicted value of optimal formula (PC6)	85.18	575.52	21.03

EE%: entrapment efficiency percentage; PS: particle size; PDI: polydispersity index; ZP: zeta potential; PC: PEGylated cerosomes..

#### Effect of formulation variables on the EE%

**Figure 1.**
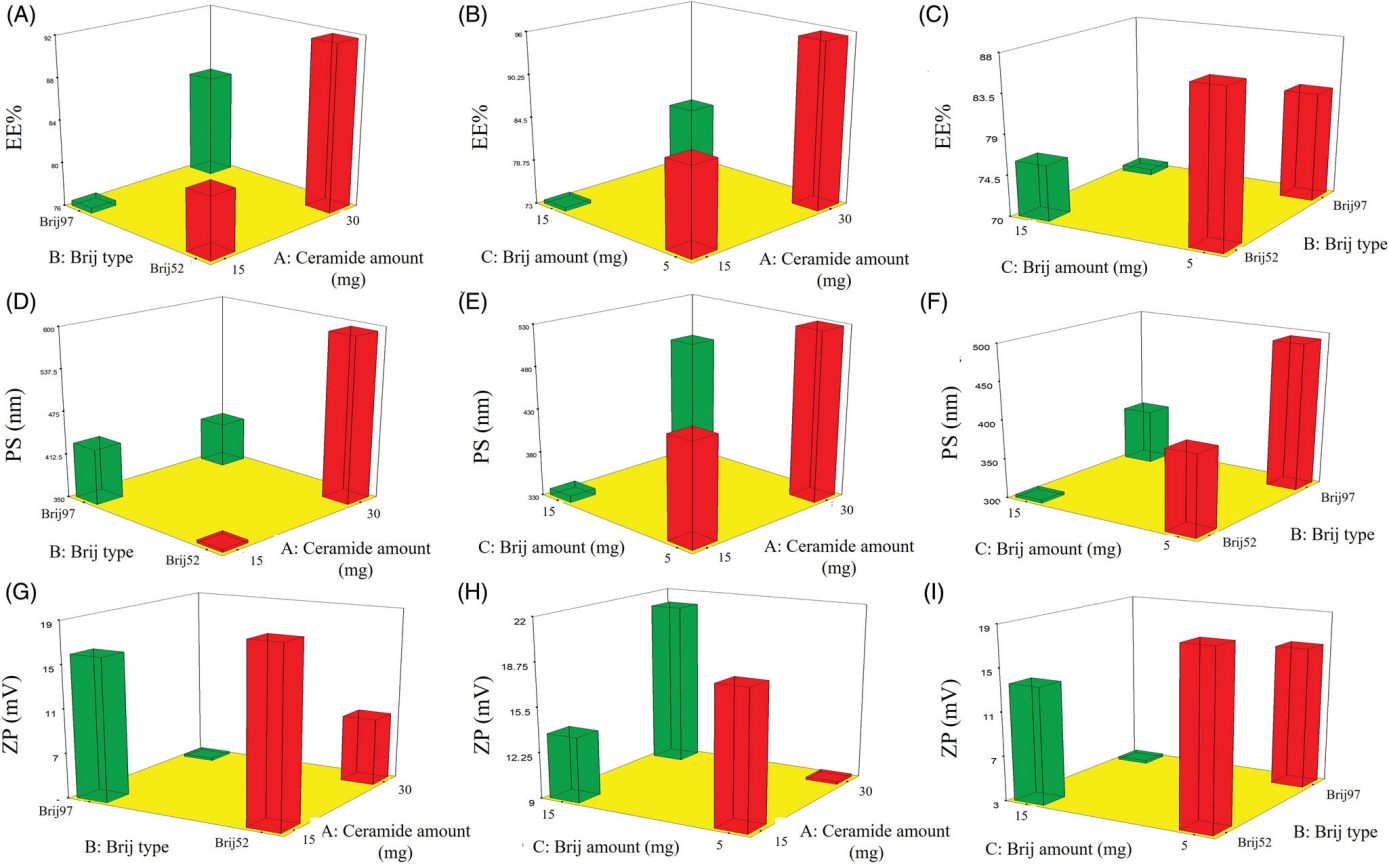
3D surface plots for the effect of ceramide amount (X_1_), Brij type (X_2_) and Brij amount (X_3_) on EE% (A-C), PS (D-F) and ZP (G-I) of FTN loaded PCs. EE%: entrapment efficiency percent; PS: particle size; PDI: polydispersity index; ZP: zeta potential; FTN: fenticonazole nitrate; PC: PEGylated cerosomes.

The ability of the formulated vesicles to entrap a reasonable quantity of drug is an important parameter for its potential application as a topical drug delivery system. In our study, the presence of the hydrophobic ceramide and phosphotidylcholine supported the high incorporation of the water-insoluble FTN molecules within the formulated PCs. As illustrated in Table 2, EE% of the formulated FTN-loaded PCs ranged from 69.66 ± 0.47 to 99.33 ± 0.48% w/w. ANOVA statistical analysis of the data revealed that all the studied variables showed a significant effect on the EE% (Figure 1(A–C)). Increasing the amount of ceramide from 15 to 30 mg in the formulation (X_1_) resulted in a significant positive effect on EE% (*p* < 0.0001). It was previously reported that increasing the amount of ceramide increases the viscosity of the formulated colloidal dispersion (Abdelgawad et al., 2017). The highly viscous formulations will hinder drug diffusion to the external aqueous phase thus ensuring higher EE% values (Yousry et al., 2019).

Also, the type of Brij (X_2_) showed a significant effect on the EE% of the formulated PCs (*p* < 0.0001). It was found that PCs formulated using Brij52 showed higher FTN entrapment when compared to Brij97. This finding might be related to the HLB values of both surfactants (5.3 and 12.4 for Brij52 and Brij97, respectively) (Tagami et al., 2011). Surfactants with low HLB values display more lipophilicity and better suitability for incorporation of hydrophobic drugs like FTN and thus, higher EE% was observed with vesicles formulated using Brij52 rather than Brij97. On the other hand, the unsaturated acyl group in Brij97 molecular structure may be an additional factor for its lower EE%. The presence of unsaturated double bond in the carbon chain might cause chain twisting which may result in a more relaxed molecules packing and loose vesicle bilayer that prompts drug leakage (Bnyan et al., 2018). In addition, the statistical analysis showed a significant negative effect of Brij amount (X_3_) on the EE%, (*p* = 0.0004) where increasing Brij amount from 5 mg to 15 mg resulted in PCs with lower EE%, This effect was anticipated due to the introduction of more permeable pores in the vesicle bilayer and the increase in the membrane fluidity with the increase in the surfactant molecules which permit drug leakage and lower EE% values (Al-Mahallawi et al., 2014; Bnyan et al., 2018).

#### Effect of formulation variables on PS

The small particle size of the nanodispersion is important to produce a kinetically stable system that resists particle aggregation and/or sedimentation. In addition, the particle size of the nanosystem may affect drug retention as well as the extent of its skin permeation (Roberts et al., 2017). As depicted in Table 2, all the formulated PCs showed a small PS range that extended from 309.20 ± 9.06 to 630.00 ± 9.20 nm. Statistical analysis of the PS data by ANOVA showed that all the studied factors affect the PS significantly as shown in Figure 1(D–F). The increase in the amount of ceramide (X_1_) incorporated in the formulation resulted in vesicles of larger PS (*p* < 0.0001). It was previously reported that increasing ceramide amount tend to form aggregates with subsequent PS enlargement. One of the hypotheses that have been proposed to explain ceramide-induced structural alterations was based on the low ability of ceramide to cross between membrane leaflets. Accumulation of ceramide in the leaflet would promote changes in membrane curvature and consequent PS enlargement (Castro et al., 2014). In regard to Brij type (X_2_) (*p* < 0.0001), the PS of the formulae containing Brij52 was significantly higher compared to Brij97 formulae. This can be explained in terms of the higher EE% observed with Brij52 as previously reported by Yousry et al. (2017). In addition, the PEG content of both surfactants (2 and 10 PEG units in Brij52 and Brij97 units, respectively) might be another reason for the effect of Brij type on the PS of the formulated vesicles. It was reported that the decrease in PEG content of the PEGylated surfactant might increase the rate of vesicles’ precipitation and vesicles’ agglomeration resulting in higher PS (Caliceti et al., 2004). Finally, increasing the Brij amount (X_3_) significantly decreased the PS of the formulated vesicles (*p* = 0.0019). This effect is anticipated due to the surface-active property of Brij which reduces the interfacial tension of the system and augments the curvature of vesicles resulting in lower PS at a higher Brij amount. Whereas at a low level of Brij, the surfactant amount might be insufficient to effectively cover the lipids and reduce the surface tension, and hence, larger vesicles were formed (Zeb et al., 2016). In addition, a larger amount of surfactant can enhance the stability of the formulated systems due to the formation of a steric barrier on the surface which hinders aggregation of particles (Yousry et al. 2016).

#### Effect of formulation variables on PDI

PDI values of the dispersion indicate the homogeneity and quality of the formulated nanosystem. A PDI of low value approaching zero represents homogenous dispersion with a narrow PS range. On the other hand, a value approaching 1 indicates highly polydisperse dispersion (Albash et al., 2019). In our study, the PDI values of all the formulated systems ranged from 0.44 ± 0.05 to 0.84 ± 0.03 (Table 2) which indicated the polydispersity of some of the prepared PCs (Stetefeld et al., 2016). These high PDI values might be a result of the irregular tubulated vesicular shape of cerosomes; however, only systems with a narrow range of PS were included in the optimization step. All the inspected factors; ceramide amount (X_1_), Brij type (X_2_) and Brij amount (X_3_) revealed a non-significant effect on the PDI of the systems with *p* values of 0.91, 0.21 and 0.38, respectively.

#### Effect of formulation variables on ZP

ZP measurement is used to identify the total surface charge of the formulated nanodispersion to determines its physical stability and anticipate any possible interaction within the body (Auría-Soro et al., 2019). Human skin carries negative charges on its surface due to the presence of negatively charged residues of proteins (Fang et al., 2004). Thus, a positively charged delivery system will strongly interact with the surface of the skin resulting in better drug retention in skin layers. In addition, the higher absolute values of ZP indicate higher surface charge, lower particle-particle interaction and better physical stability of the formulated vesicles (Radwan et al., 2020).

As shown in Table 2, all PCs showed positive ZP values that ranged from 2.74 ± 0.13 to 20.90 ± 0.55 mV. The positive ZP values observed with the formulated vesicles are correlated with the addition of SA which induced positive charge dominating over the other excipients used (Al-Mahallawi et al., 2014; Radwan et al., 2020). Upon statistical analysis of data, ANOVA test revealed that both the amount of ceramide and Brij type significantly affected ZP values as shown in Figure 1(G–I). Increasing the amount of ceramide (X_1_) resulted in significantly lower values of ZP (*p* < 0.0001). Ceramide IIIB possesses an amphiphilic structure that might deposit on the surface of the prepared vesicles shielding the positive charge of the formulated PCs and resulting in lower ZP values (Yilmaz & Borchert, 2005). On the other hand, FTN-loaded PCs formulated using Brij97 showed significantly lower ZP values when compared to those formulated using Brij52 (*p* < 0.0001). These lower values of ZP might be attributed to the higher number of the PEG units found in Brij97 (10 units) compared to Brij52 (2 units). It was previously reported by Hu et al (Hu et al., 2007) that the presence of the hydrophilic PEG molecules may form a steric barrier that screens the charge at the surface of the vesicles resulting in lower ZP values.

### Selection of the optimal FTN-loaded PC

The optimum levels of the independent variables were obtained by analysis of the data of dependent variables using Design Expert^®^ software in order to prepare an optimal FTN loaded PCs system with the highest EE% and ZP values with the lowest possible PS and PDI. The software selected PC6 as an optimal formula with the highest desirability factor (0.733). The selected formula was prepared using 30 mg of ceramide, and 15 mg Brij52. It showed nanosized vesicles (551.60 ± 23.84 nm) with high FTN EE% of 83.00 ± 1.63% w/w, and an acceptable ZP value of 20.90 ± 0.55 mV. The predicted values of the dependent variables for optimal FTN-loaded PC ‘PC6’ were in good agreement with the actual values (Table 3) indicating the suitability of the statistical design for the statistical evaluation and analysis of the different formulation variables (Yousry et al. 2020).

#### Transmission electron microscopy (TEM)

**Figure 2.**
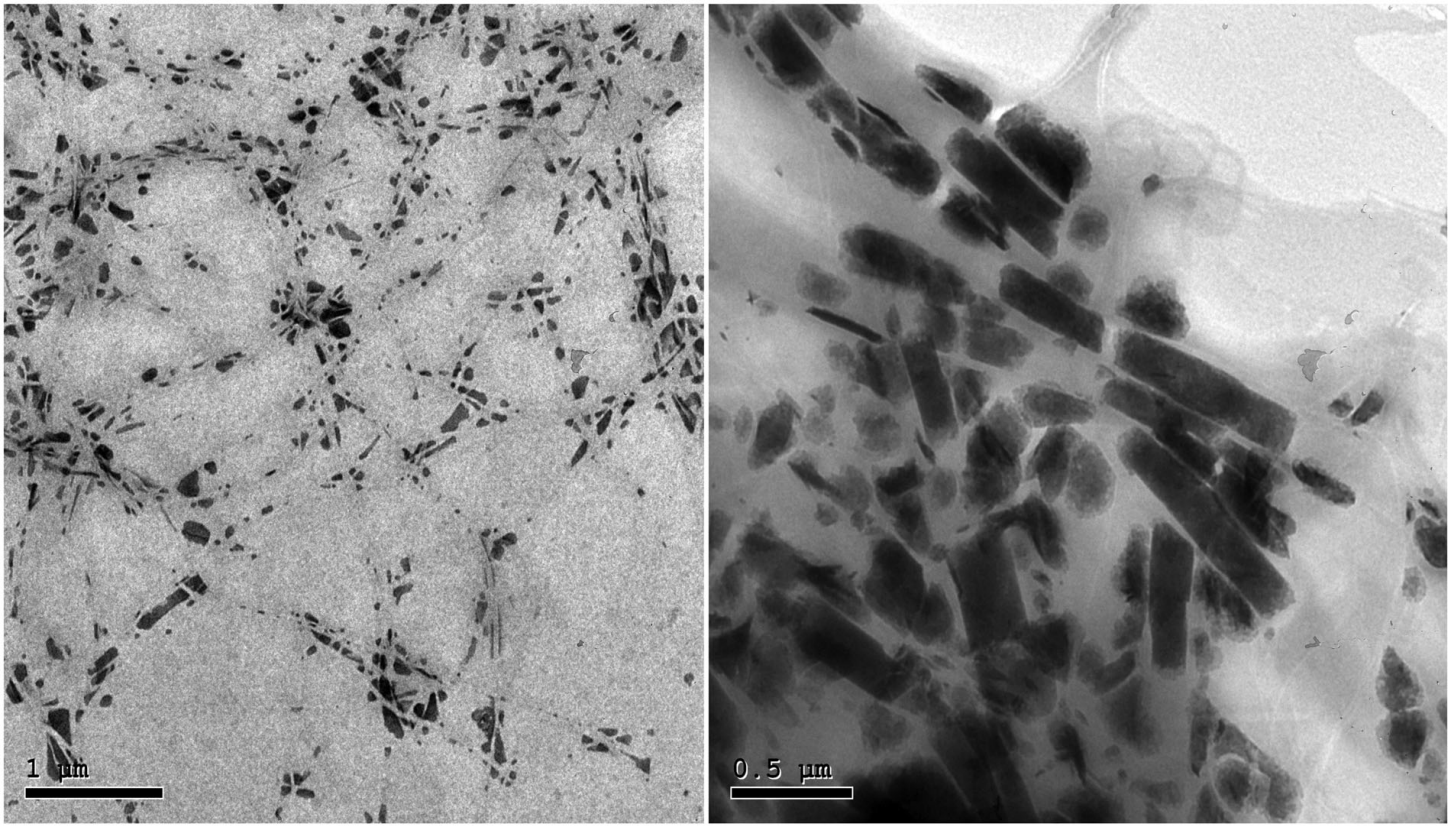
Transmission electron micrograph of the optimal FTN-loaded PEGylated Cerosomes (PC6).

The transmission electron micrograph of the optimal FTN-loaded PC (PC6) shown in Figure 2 demonstrated the distinctive fiber-like shape of cerosomes with the formation of elongated intertwined ceramide tubules. These images were in a good agreement with those previously observed by Abdelgawad et al. (2017).

#### Short term physical stability study

The optimal formula (PC6) was subjected to short-term stability study for 3 months. The visual inspection of the stored system did not show any sedimentation or vesicles’ aggregation during the storage period. In addition, PS, PDI and EE% measurements of the stored optimal formula were 580.9 ± 35.46 nm, 0.466 ± 0.103 and 87.80 ± 2.85% w/w which showed insignificant variation from the freshly prepared system (*p* > 0.05). These findings revealed the physical stability of the formulated cerosomes which may be related to the steric stabilization created by the PEGylated surfactant (Abdelbary et al., 2019).

### In-vivo *studies*

#### Dermatokinetic study

**Figure 3.**
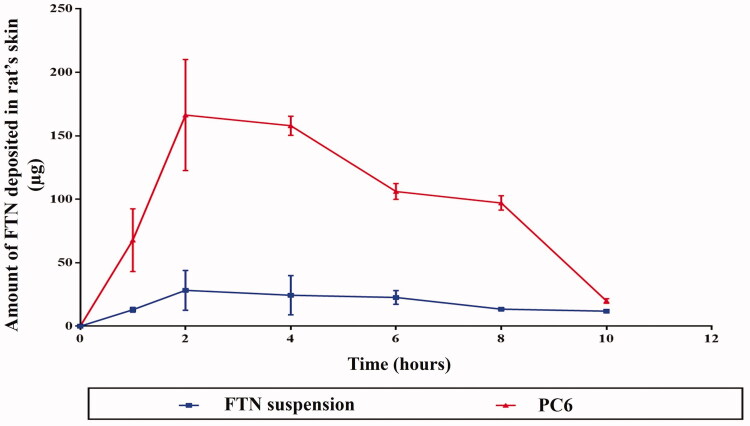
Mean (±SD) skin-FTN concentration following topical application of the optimal FTN-loaded PC (PC6) and FTN suspension to 18 Wistar rat each. FTN: fenticonazole nitrate; PC: PEGylated cerosomes. Data represented as mean ± SD (*n* = 3).

The dermatokinetic study was performed to estimate the amount of FTN deposited in the skin layers at different time intervals after application of FTN suspension and the optimal FTN-loaded PCs ‘PC6’ (Moolakkadath et al., 2018). As illustrated in Figure 3, the skin treated with the optimal PC6 showed higher drug concentration level in the skin at all time intervals with significantly higher C_max_ of 172.02 ± 22.86 μg/cm^2^ and AUC_0–10_ of 1120.61 ± 33.97 μgh/cm^2^ compared to 34.34 ± 5.60 μg/cm^2^ and 306.27 ± 32.18 μgh/cm^2^, respectively, with FTN suspension (*p* < 0.05). However, there was no significant difference between the T_max_ of both systems. The higher levels of FTN in the skin at the different time intervals suggest the localization of the optimal PC system within the skin layers. This localization can be attributed to the addition of Brij as PEGylated surfactant where it was previously reported by Vega et al. (2013) that PEG presence within nanoparticle formulation might modulate the interfacial characters of the nano-system and influence drug deposition which prolong their residence time at the site of action. On the other hand, the ceramide-content in PCs is known for its ability to interact with the keratin layer of the corneocytes, permitting lipid bilayer holding behavior and high fusion activity to the skin resulting in drug localization (Abdelgawad et al., 2017).

#### Histopathological study

**Figure 4.**
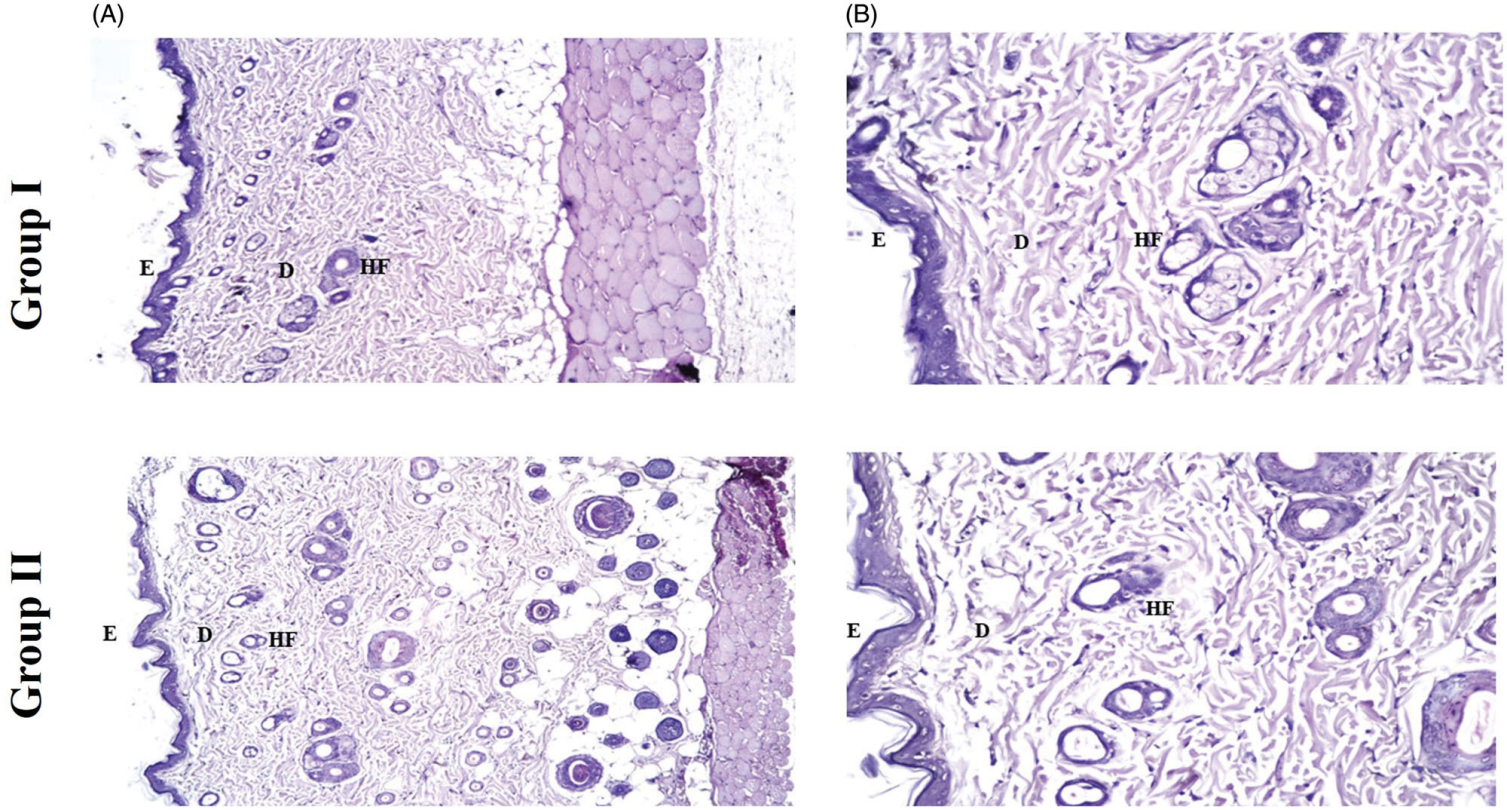
Photomicrographs showing histopathological sections (hematoxylin and eosin stained) of rat’s skin normal control (group I) and rat’s skin treated with PC6 (group II) with magnification power of 16x to illustrate all skin layers (A) and magnification power of 40x to identify the epidermis and dermis (B). E: epidermis; D: dermis; HF: hair follicles; FTN: fenticonazole nitrate; PC: PEGylated cerosomes.

*In-vivo* histopathological study was conducted to observe any structural changes in the skin of rats after exposure to the optimal FTN-loaded PCs (PC6) three times daily for one week and comparing it to another control group of rats. The light microscopy examination of both groups shown in Figure 4 revealed that PC6 (group II) did not show any histopathological alternations in the epidermal and dermal cells of the rats’ skin when compared to untreated skin sections (group I). These findings support the safety and tolerability of PEGylated cerosomes for topical application in skin infections.

## Conclusion

Topical application of anti-fungal agents is favored over oral therapy in terms of avoiding the risk of systemic side effects, in addition to its selective ability in targeting the site of infection. In the current manuscript, the utilization of PCs was proven to achieve enhanced localization and topical delivery of FTN. A 2^3^ full factorial experimental design was adopted to study the formulation variables and select the optimal FTN-loaded PCs (PC6). PC6 was successfully formulated as nano-sized tubulated vesicles with small PS, high EE% and acceptable ZP values. The *in-vivo* dermatokinetic study confirmed the retention tendency of FTN-loaded PCs in rat’s skin compared to FTN suspension. In addition, the histopathological study confirmed the tolerability and safety of PC6 multiple applications. In the consequence, the previous findings proved that PEGylated cerosomes could be a potential delivery system for topical application of FTN.
